# Development of an in vivo protocol for investigating natriuretic mechanisms in response to acute sodium loading in humans

**DOI:** 10.1186/1753-6561-6-S4-O45

**Published:** 2012-07-09

**Authors:** LA Phillips, IM MacIntyre, DJ Webb

**Affiliations:** 1 University of Edinburgh, UK

## Introduction

Extracellular fluid volume and arterial blood pressure are principally determined by total body sodium via osmoregulation. Dysregulation of mechanisms involved in maintaining sodium balance are therefore implicated in the development of hypertension. Hormonal mechanisms are thought to be critically involved in the regulation of natriuresis, and modification of these mechanisms may be a useful strategy in managing hypertension. Our aim was to establish an appropriate protocol for inducing natriuresis, which could be used to investigate the role of various hormone systems in maintaining sodium balance, and the effects of using pharmacological agents to manipulate these systems.

## Methods

0.9% Saline was administered to 6 healthy subjects at a rate of 500ml/hour over a period of two hours.

## Results

Significant natriuresis occurred within 2 hours of sodium loading (p<0.01) and was induced independently of peripheral blood pressure.

## Conclusions

By demonstrating that significant natriuresis can occur within the timeframe of our protocol, and that it is independent of non-hormonal mechanisms such as pressure natriuresis, we have developed a protocol that can be applied to the investigation and subsequent manipulation of hormonal control of natriuresis. We aim to use this protocol to study the role of the endothelin system in natriuresis.

